# Cell Therapy Using Anti-NKG2A Pretreated Natural Killer Cells in Patients with Hepatocellular Carcinoma

**DOI:** 10.34172/apb.43869

**Published:** 2024-12-05

**Authors:** Shirin Tavakoli, Maryam Samareh-Salavati, Shahrokh Abdolahi, Javad Verdi, Iman Seyhoun, Nasim Vousooghi, Mohammad Vaezi, Afshin Ghaderi, Ardeshir Ghavamzadeh, Maryam Barkhordar, Mohammad Ahmadvand

**Affiliations:** ^1^Department of Applied Cell Sciences, School of Advanced Technologies in Medicine, Tehran University of Medical Sciences, Tehran, Iran.; ^2^Basic and Molecular Epidemiology of Gastrointestinal Disorders Research Center, Research Institute for Gastroenterology and Liver Diseases, Shahid Beheshti University of Medical Sciences, Tehran, Iran.; ^3^Cell Therapy and Hematopoietic Stem Cell Transplantation Research Center, Research Institute for Oncology, Hematology and Cell Therapy, Tehran University of Medical Sciences, Tehran, Iran.; ^4^Department of Internal Medicine, Hematology and Medical Oncology Ward, Yasuj University of Medical Sciences, Yasuj, Iran.

**Keywords:** Natural killer cells, Hepatocellular carcinoma, NKG2A, Inhibitory receptor

## Abstract

**Purpose:**

The activities and functions of natural killer (NK) cells are regulated by a limited repertoire of activating and inhibitory receptors. Thus, we provided a study of inhibition of the NKG2A using monoclonal antibodies (mAbs), and as a primary endpoint, we evaluated whether it can be translated to enhance adoptive NK cell immunotherapy, as the secondary endpoint, we investigated safety and feasibility.

**Methods:**

In this study, we investigated the safety of anti-NKG2A-pretreated NK cells in improving ADCC function to manage hepatocellular carcinoma (HCC). After a conditioning regimen, we initiated a pilot study of expanded donor haploidentical NK cell infusion. Patients received a fludarabine/cyclophosphamide conditioning followed by adoptive immunotherapy with IL2–activated haploidentical NK cells. Anti-NKG2A pretreated NK cells were infused on days 0,+5, and+10 post-conditioning regimens at a dose of 7×10^8^ cells (n=3). The median follow-up was 4 months for all patients.

**Results:**

Although all patients were alive at the last follow-up, two of them showed progressive disease and an increase in tumor size. In addition, all patients showed a relative decrease in alpha-fetoprotein (AFP) expression levels after one month.

**Conclusion:**

This study demonstrated the safety and feasibility of infusing high doses of ex vivo expanded NK cells after conditioning with transient side effects.

## Introduction

 Hepatocellular carcinoma (HCC) ranks as the sixth most common cancer worldwide and is expected to cause the death of 1.3 million people in 2040.^1^ Based on annual estimates, HCC is known as the most common type of liver-related cancer, 75%-85%, which has a 5-year survival rate of less than 20% and an incidence-to-mortality ratio close to 1.^2,3^ The most unresectable HCC patients are diagnosed in advanced stages, which limits treatment options.^4^ In this regard, the development of targeted systemic therapies has been promising.^5-7^ Sorafenib as a tyrosine kinase inhibitor and Lenvatinib are considered first-line therapy.^8,9^ Their response rate is unsatisfactory, with a median overall survival of 13.6 months.^10^ Hence, the investigation of novel treatment strategies has become critical. Research has increasingly focused on developing immune cell-based therapies for HCC since the first approval of this type of therapy. Therefore, numerous research studies have investigated the potential of immune cells for therapeutics.^11,12^

 Natural killer (NK) cells have been proposed as a promising immune cell-based therapy in treating solid tumors.^12,13^ NK cells directly or indirectly perform their antitumor activity to control tumor invasion through immune surveillance mechanisms. Their activation is provoked by competition between activating and inhibitory receptors. In addition, their regulatory function is important.^14,15^

 Among the benefits listed for NK cells, less chance of causing graft-versus-host disease (GVHD) and reduced risk for induction of a potentially lethal cytokine release syndrome have caused NK cells to be promising as “off-the-shelf” allogeneic products.^16,17^ Despite the great potential of NK cells, several reports have shown dysfunction of adaptive NK cell monotherapy and weak infiltration in solid tumors.^18-20^ There are several reasons why NK cell in this approach is inhibited in the tumor microenvironment, such as the reduction of natural cytotoxicity receptors, shedding of major histocompatibility complex class I chain-related protein A and B, known as the ligands of NKG2D, stimulating immune checkpoints on the NK cells, and the overexpression of inhibitory receptors which causes NK cell exhaustion at the tumor site.^14,21^ Addressing the limitations above is crucial to enhancing the efficacy of NK cell therapy. Considering NK cell checkpoints has opened new horizons that have a key function in increasing the toxicity of these cells.

 NKG2A is a cell surface molecule complexed with CD94 and forms an inhibitory receptor in NK cells.^22-24^ NKG2A plays an essential regulatory role when it binds to its ligand (non-classical HLA class I molecule HLA-E).^22^ In this context, overexpression of this ligand has been reported in various malignancies, such as HCC, which has been linked with a worse prognosis.^25-27^ In addition, the upregulation of NKG2A on NK cells allows cancer cells to bypass immune surveillance and facilitate the process of metastasis.^28^ Furthermore, half of blood NK cells express NKG2A, which is more reactive towards transformed HLA class I negative target cells.^29^ Several studies have shown that blocking the binding of NKG2A to its ligand, which is expressed in cancer cells, significantly improves immunotherapy results, so NKG2A is considered a checkpoint for NK cells.^30,31^

 Monalizumab (anti-NKG2A) is a monoclonal antibody designed to target NKG2A receptors on NK cells. By blocking the inhibitory signals from these receptors, Monalizumab helps to release the brakes on the immune system, enhancing its ability to attack cancer cells. This mechanism has shown potential in treating certain highly immunogenic tumors, empowering the immune system to respond more effectively against cancer.^32,33^ Limited research has been conducted on adaptive NK therapy for HCC, in which a partial response of 18% has been shown^34^ In this study, we investigated a combination therapy of activated NK cells pre-treated with anti-NKG2A, an NKG2A blocker, to evaluate its safety in HCC patients.

## Materials and Methods

###  Eligibility

 Three patients with histologically confirmed diffuse form HCC who were unresectable, locally advanced, and/or metastatic were enrolled in this pilot clinical study between March 2024 and May 2024 after completing the consent form. Eligible patients, 60 < age > 20 years, previously standard therapies failed, having the same tumor size and prior treatment protocol, Eastern Cooperative Oncology Group (ECOG) performance status ≤ 2, life expectancy of at least 3 months, not having serious cardiovascular disease, adequate vital organ function, and normal blood test results as leukocyte count ≥ 3000/mm^3^ and ≤ 12 000/mm^3^, neutrophil count ≥ 1500/mm3, platelet count ≥ 100 000/mm^3^, hemoglobin ≥ 9.0 g/dL, serum aspartate aminotransferase (AST) and alanine aminotransferase (ALT) ≤ 100 IU/L, serum total bilirubin ≤ 2 mg/dL, serum creatinine (Cr) ≤ 1.5 mg/dL, and blood urea nitrogen (BUN) level ≤ 25 mg/dL. The exclusion criteria were as follows: positive record for hepatitis B or C virus (HBV or HCV), human immunodeficiency virus (HIV), HTLV-1, or syphilis infection, other active serious infection, serious complications such as severe diabetes mellitus, unstable angina, or myocardial infarction within 3 months, not pregnant or breastfeeding, a medical history of severe hypersensitivity or autoimmune disease, or had received any type of cell therapy in the 6 months before inclusion.

###  Study design

 This pilot clinical study was a nonblinded and nonrandomized study and was approved by the ethics committee of the Tehran University of Medical Sciences (ethics code: IR.TUMS.TIPS.REC.1403.015). Also, it was registered in the Iranian clinical trial data set (IRCT) as ID: IRCT20140818018842N41on June 15, 2024. Patients were chosen randomly for dose administration, and their enrolment was carried out according to chronological order established at the project’s start. All participants were familiar study requirements. This multi-central study was designed and conducted by the Cell Therapy and Hematopoietic Stem Cell Transplantation Research Center of Shariati Hospital and the Department of Applied Cell Sciences of the School of Advanced Technologies in Medicine of Tehran University of Medical Sciences. NK cell preparation, patient enrollment, and clinical steps were performed at the Department of Oncology of Shariati Hospital. The primary expected outcome of this project was the feasibility and safety of the adoptive transfer of allogenic anti-NKG2A pretreated NK cells in patients with HCC, and the secondary endpoint was clinical anti-tumor feedback.

###  Cell processing

 After signing informed consent, their blood was checked for the contamination of HBV, HCV, and HIV tests. Based on the previous protocol 35, whole blood (20 mL from each donor) was taken from random healthy unrelated donors by intravenous blood sampling using blood collection syringes and injectors in tubes with ethylenediaminetetraacetic acid (EDTA). After collection, samples were immediately transferred to a clean room under cold conditions to isolate peripheral blood mononuclear cells (PBMCs). PBMCs were isolated using Ficoll Paque Premium (GE Healthcare’s, United States) density gradient centrifugation for 20 minutes at 300 g. After separating the final mixture into three layers, the middle layer was carefully collected using a pipette, moved to a different tube, and given two washes with PBS that had been preheated to 25 °C. The cell pellets were re-suspended in 1 milliliter of RPMI1640 (Gibco, USA). The cell viability was then visually evaluated and counted using 4% trypan blue dye (Sigma-Germany).

 Highly purified NK cells were obtained according to the manufacturer’s instructions (Miltenyi Biotec, Germany), and non-NK cells were depleted through a magnetically activated cell sorting (MACS) system. NK cell purity was evaluated by flow cytometry using anti-human CD3 and CD56 monoclonal antibodies (mAbs). For NK cell expansion, isolated cells were seeded at 5 × 10^5^ cells/mL and cultured with irradiated (100 Gy) k562-genetically modified cell line (5 × 10^6^ cells/mL) in a T-25 culture flask. The culture medium was RPMI1640 (Gibco-USA) containing 1000 IU/mL IL-2 (IL-2, Miltenyi Biotec-USA), 10 ng/mL IL-15, and 5% of human serum (Sigma-Aldrich). On day 5, expanded NK cells were transferred to a bigger flask in the RPMI medium containing 1000 IU/mL IL-2, 10 ng/mL IL-15, and 5% of human serum. A fresh culture medium containing 1000 IU/mL IL-2, 10 ng/mL IL-15, and 5% of human serum was added to the flask every 2 to 3 days for 21 days. Procedures for NK cell expansion were carried out under GMP principles.

###  Quality control and characterization of isolated NK cells

 The acceptance criteria for all NK cell products at the end of the process included bacterial and endotoxin contamination assessment using Gram staining and a kinetic turbidimetric limulus amebocyte lysate (LAL) assay, respectively. Fungal and mycoplasma contamination was detected by culture using a MycoAlert Mycoplasma Detection Kit (Lonza Japan, Tokyo, Japan). The trypan blue exclusion assay was used to assess cell viability. The karyotype of the cells was checked after treatment with anti-NKG2A antibody. The purity of peripheral blood-derived NK cells (PB-NK) was determined by quantitatively assessing the ratios of CD3^-^ and CD56^+^ markers using flow cytometry (FACSCalibur Becton Dickinson, USA). Briefly, a total of 1 × 10^5^ cells were collected, washed with phosphate buffer saline (PBS), and stained with fluorochrome-labeled antibodies (CD56-APC and CD3-FITC, Biolegend, USA) for 20 min at room temperature and in the dark. The cells were washed, and the proportion of labeled cells was determined using a flow cytometry instrument and FlowJo software. To evaluate the in vitro cytotoxic effect of human-activated NK cells (effector cells), the K562 cell line was chosen as the target cell line. The effector (E) and target (T) cells were co-cultured in ratios of 1:1, 5:1, and 10:1, followed by measuring the presence of lactate dehydrogenase (LDH) in the cell culture medium after 24 h. The frequency of LDH is directly associated with the necrosis and cytotoxicity of NK cells. Activated NK cells were co-cultured with the K562 cell line for 24 hours, and the supernatants were collected. Following the manufacturer’s instructions, ELISpot kits (Sigma Aldrich, USA) were used to measure the levels of IFN-γ and TNF-α.

###  Pretreatment of NK cells with anti-NKG2A

 First, the culture medium was centrifuged at 500 g for 5 minutes to isolate NK cells. Following the assessment of NKG2A abundance on the NK cell surface, the cells were treated with 4 mg/mL anti-NKG2A antibody for 20 minutes. A decrease in the expression of the NKG2A receptor was examined by flow cytometry and LDH assay.

###  Schedule of administration and dosages

 The expanded cells were pooled into labeled cell infusion bags containing 50 mL of normal saline (9%) and albumin (5%), which were transported at 4 °C–8 °C to the Department of Oncology of Shariati Hospital. Over 40 minutes, the patients received intravenous infusions of fresh PB-NK cells (7 × 10^8^ cells). The infusion was repeated every five days for three cycles, and during the first and fourth months, the patients were monitored, respectively.

###  Patient follow-up and laboratory results

 To assess safety and toxicity, regular patient interviews, examination of clinical conditions, and vital signs were evaluated by the principal investigator. Further, hematologic and biochemical parameters were checked. For this purpose, the National Cancer Institute Common Terminology Criteria for Adverse Events (CTCAE, version 5.0) checklist was applied. It focuses on parameters like anaphylactic shock, heart attack, variation in lung capacity, fever, and rash. These symptoms were recorded using remote patient monitoring (RPM) devices from the beginning of infusion to discharge for 1 month. In addition, computed tomography (CT) scans were analyzed based on the Response Evaluation Criteria in Solid Tumor (RECIST VERSION 1.1) to evaluate tumor response before the start and 1 and 4 months after the end of cell therapy.

 The number of blood cells containing white blood cells (WBCs), red blood cells (RBCs), and platelets were counted before cell therapy (at the hospitalization date) and 48 hours after the last cell infusion via an automated cell counter device (Sysmex, Japan). Furthermore, biochemical markers, including alkaline phosphatase (ALP), ALT, BUN, Cr, and C-reactive protein (CRP), were measured before the initial cell injection and 48 hours after the last cell infusion using related kits. Importantly, the alpha-fetoprotein (AFP) tumor marker level was measured three times: before the beginning of cell therapy and 1 and 4 months after the end.

###  Statistical analysis

 Statistical analysis of data gathered pre- and post-NK cell combination therapy was performed using the Wilcoxon signed-rank test, with a significance threshold set at *P* < 0.05, employing GraphPad Prism 5 for Windows for the evaluations.

## Results and Discussion

###  Patient characteristics

 The limited number of NK cells in the blood circulation and the tumor area and their impaired cytotoxic potential have caused the therapeutic potential of these cells to be considered for HCC patients.^35,36^ Despite the confirmed benefits of adoptive NK cell therapy, tumor site suppression and tumor infiltration are still obstacles.^37,38^

 From March 2024 to May 2024, three eligible patients with similar tumor sizes (one female and two male) were enrolled in this pilot study. The complete attributes of patients are listed in Table 1. The mean age of enrolled individuals was 53 years (range: 49-59). All patients showed evidence of lung and lymph node metastases, and the median size of the tumor was substantial ( > 5 cm). All three subjects had received the same chemotherapy regime before the trial with no history of radiation therapy. During the administration period, none of the patients showed any evidence of disease progression and all of them completed their infusions.

**Table 1:** Patient characteristics

**Case**	**Age/Gender**	**Diagnosis**	**ECOG**	**Disease Stage**	**Prior treatment**	**NK cell administration**
1	51/M	Hepatocellular carcinoma	≤ 2	Metastasis to lungs, lymph nodes	Sorafenib	Completed
2	59/M	Hepatocellular carcinoma	≤ 2	Metastasises to lungs	Sorafenib	Completed
3	49/F	Hepatocellular carcinoma	≤ 2	Metastasises to lungs	Sorafenib	Completed

###  Characterization of expanded NK cells

**Figure 1:**
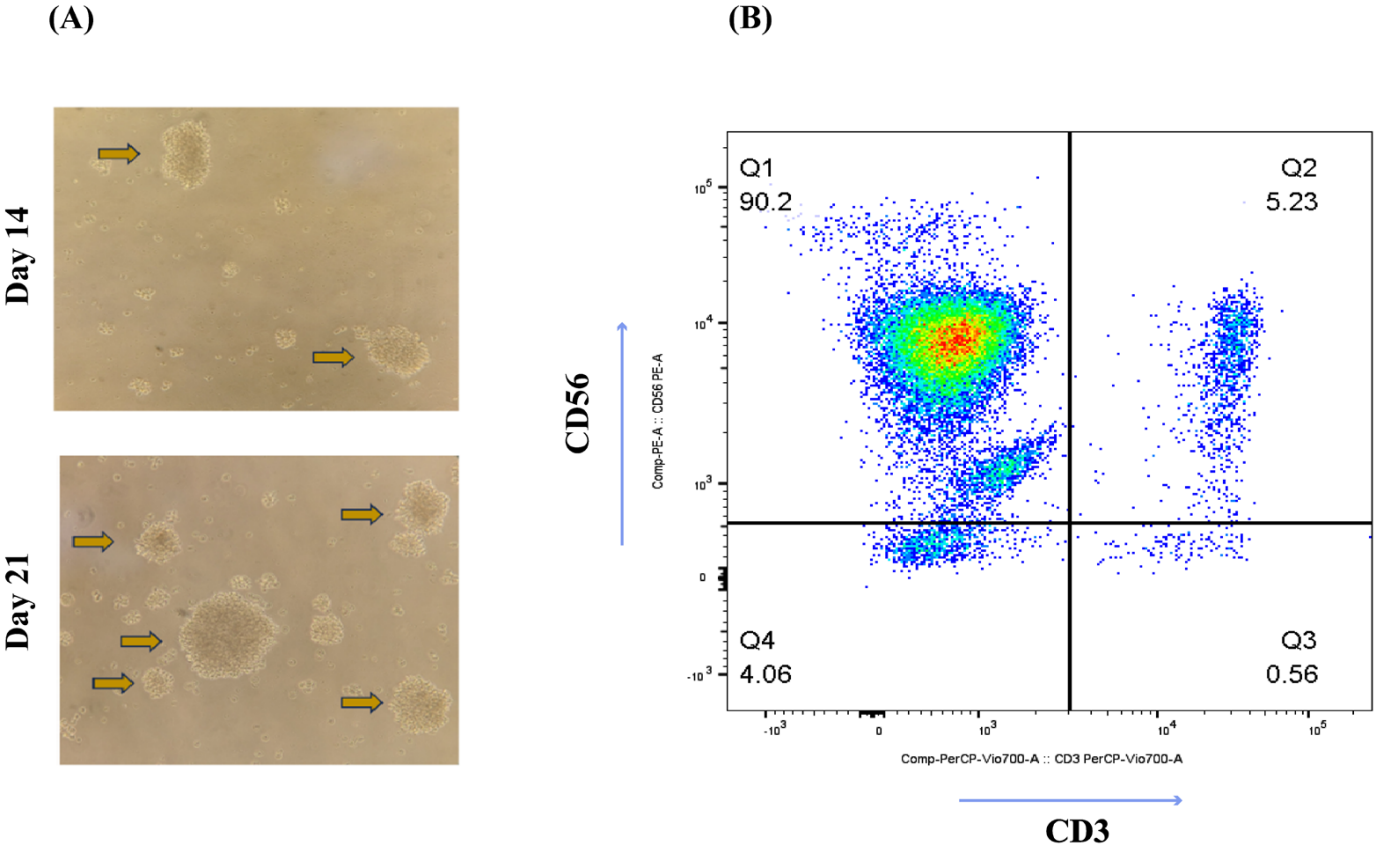
Human NK cell isolation and characterization. (A) NK cells exhibited round colonies as depicted by the arrows after 14 and 21 days of cultivation. (B) CD56 + CD3- PBMC-derived NK cells' frequency was 90.2% after MACS isolation

**Figure 2:**
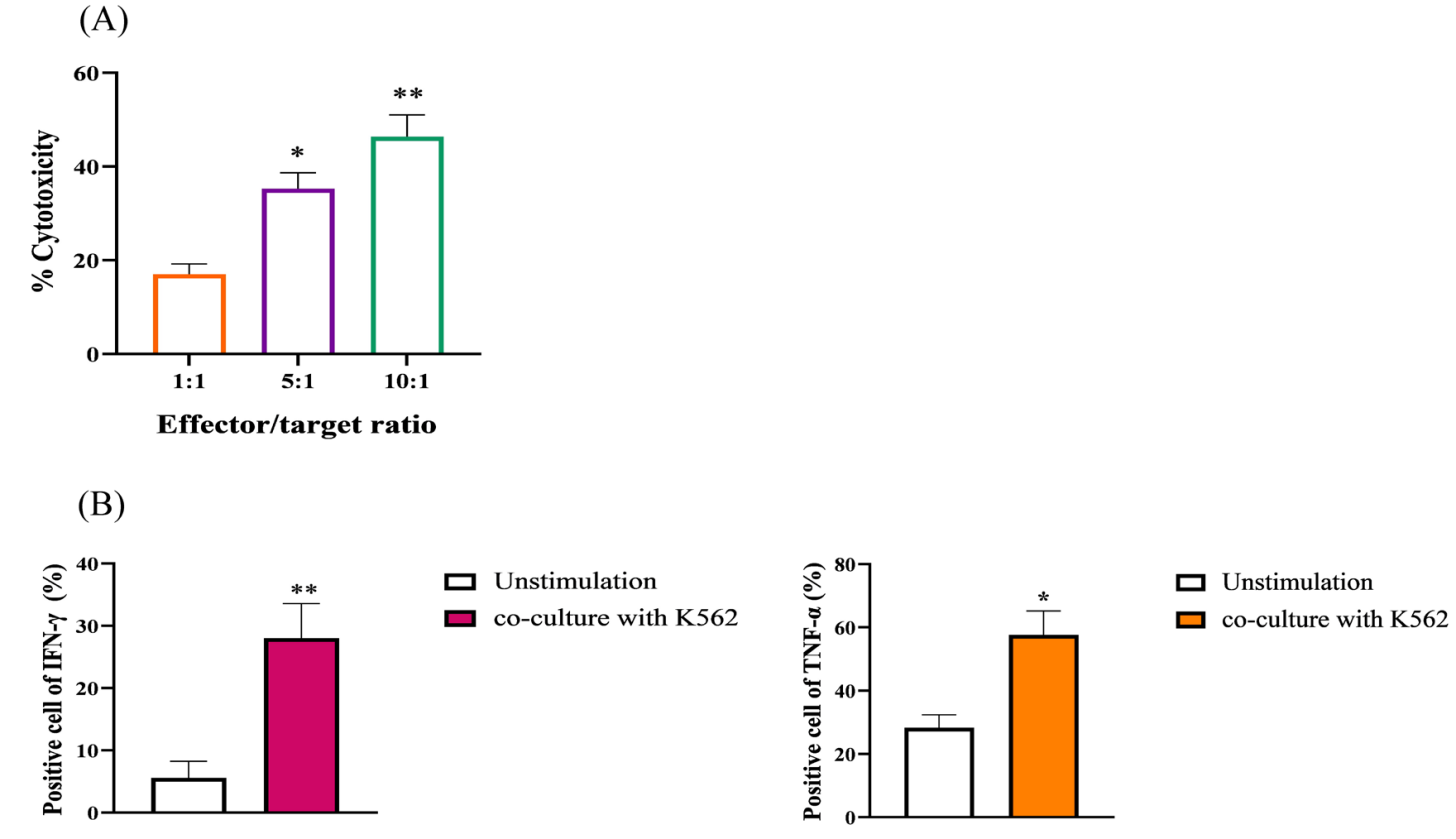
Cytotoxicity and cytokine release of NK cells. (A) Co-culturing of activated NK cells (E) and K562 cells (T) at different E: T ratios indicated the most cytotoxic effect in 10:1 E: T ratio. (B) On day 21, the assessment of cytokine release showed that the level of IFN-γ and TNF-α was significantly increased in NK cells that cocultured with the K562 cell line, compared to the unstimulated cells. All data are presented as means ± standard deviation (M ± SD, n = 3). *P ≤ 0.05, and **P ≤ 0.01

 Culturing of separated PBMCs showed a median number of 12.34% (range 5.23–16.58) NK cells in total lymphocytes that yielded an expansion rate of 586-fold (range 105-1143) after 21 days. Clonal growth and round morphology were consistently seen from day 5 to day 21 (Figure 1A). The flow cytometry analysis of NK cell biomarkers (CD56^+^ and CD3^-^) indicated 90.2% (88.12-94.4) purity of expanded cells (Figure 1B). Additionally, trypan blue staining was used to evaluate the vitality of the cells, which showed over 90% viability before injection. The cytotoxicity test of expanded cells against the NK-sensitive K-562 cell line demonstrated strong cytotoxic function (Figure 2A). The cytotoxicity of various ratios was increased by enhancing the concentration of effector cells. The strongest cytotoxic activity of NK cells was observed in the 10:1 group with a significant statistical difference (*P* < 0.001). Following the coculturing of PB-NK cells with the K562 cell line, the suspension was removed and the level of cytokine release (IFN-γ and TNF-α) was assessed using the ELISpot test (Figure 2B). The quality control tests manifested the lack of bacteria, fungi, mycoplasma, and endotoxins in the samples, which the reference laboratory further validated.

###  Blocking of NKG2A 

**Figure 3:**
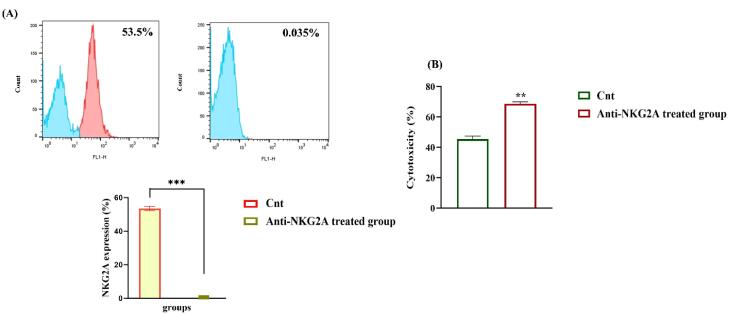
The effect of NKG2A blocking on NK cells. (A) Quantification of positive cells for NKG2A demonstrated a significant decrease in NKG2A expression following treatment of activated NK cells with anti-NKG2A Ab. (B) The cytotoxicity of anti-NKG2A-pretreated NK cells toward K562 cells at an E: T ratio of 10:1 showed a significant increase in their cytotoxicity compared with the control group. The results are displayed as mean ± SD for three independent experiments. **P ≤ 0.01, and ***P ≤ 0.001

 According to flow cytometry analysis, NKG2A expression considerably decreased from 53.5% to 0.035% after treating NK cells with anti-NKG2A (Figure 3A). Furthermore, K562 cells were co-cultured with NK cells to assess the cytotoxic effect of anti-NKG2A-pretreated human-activated NK cells *in vitro*. An LDH release assay was used to determine NK-cell-mediated cytotoxicity against K562 after a 24-hour incubation period. In the non-treated group, NK cells demonstrated 45.4% cytotoxicity, whereas in the anti-NKG2A-treated group, the cytotoxicity percentage was 68.6%. When NKG2A was inhibited using an anti-NKG2A antibody activated NK cells demonstrated remarkable anti-tumor cytotoxicity (*P* < 0.01) (Figure 3B).

 NKG2A was recently considered an ICI that is expressed by most NK cells and other cytotoxic lymphocytes.^39^ When the NKG2A receptor engages with HLA-E, intracellular phosphatase SHP-1, and SHP-2 are activated, suppressing NKG2D activation signals and inducing NK cell exhaustion.^28^ Therefore, the anti-NKG2A antibody is considered a promising checkpoint blocker that induces anti-tumor immunity by augmenting the activities of NK cells,^32^ but NKG2A blockade demonstrated poor potential as a stand-alone treatment while the NKG2A antibody has represented synergistic effects with other immunotherapies. Recent advances in popular tumor immunotherapy are “combination”, and NKG2A, a regulator of both innate and adaptive immunity may be an important option.^32,40,41^

 NKG2A blockade induces NK Cell activation after Inhibition in different mechanisms. Normally, NKG2A binds to HLA-E. This binding transmits an inhibitory signal that reduces the cytotoxic activity of NK cells. By blocking NKG2A, the inhibitory pathway is interrupted, hindering the NK cells from receiving signals that repress their function. In addition, with NKG2A blocked, NK cells are more likely to respond to activating signals through other receptors, such as NKp30 and NKG2D. Investigations have reported that blocking NKG2A can augment NK cell cytotoxicity, particularly against cancer cells that express HLA-E​. Moreover, NKG2A blockade also induces the release of cytokines like IFN-gamma and TNF-alpha by NK cells, which are crucial for anti-tumor immunity. These cytokines support the recruitment and activation of other immune cells, creating a more comprehensive immune attack on tumor cells.​^32,33,42^

 Narni-Mancinelli et al reported that NK cell education could improve efficacy, suggesting further avenues to optimize combination therapies.^43^ The enhancement of NK cell cytotoxicity by anti-NKG2A antibody is likely due to removing inhibitory signals that suppress NK cell function.

 Our pilot clinical study considered evaluating the safety, feasibility, and therapeutic potential of a combination of NK cells with an anti-NKG2A antibody in patients with HCC. According to earlier studies, immune checkpoint inhibition could promote the proficiency of NK cell treatment by inducing cytotoxic functionality.^13,44,45^

 It has been reported that approximately 20-50% of circulating NK cells express NKG2A.^46^ Significant numbers of activated NK cells were expanded under cGMP conditions using allogeneic NK cells. The expression of NKG2A remained during the expansion process, and virtually half of the expanded NK cells were NKG2A positive. Therefore, these NK cells are sensitive to anti-NKG2A antibodies due to a partial disruption of NKG2A: HLA-E ligation would be adequate to abandon NK cell’s anti-tumor immune response.^47^ We indicated that anti-NKG2A treatment significantly declined NKG2A expression and enhanced NK cell cytotoxicity in different E: T ratios.

###  Safety assessment

 CTCAE parameters like fever, rash, heart attack, respiratory changes, and anaphylactic shock symptoms were closely monitored after 48 hours of each infusion. According to the CTCAE checklist, no severe adverse effects were observed in the patients. Transient symptoms were detected in patients, one patient experienced mild fever and respiratory distress, one patient experienced headache and mild symptoms of transient fever and chill, and one patient had mild symptoms of transient chill which all were negligible. The detailed overall toxicity is shown in Table 2. Melero et al revealed a synergistically therapeutic effect of combining activated NK cells and anti-NKG2A antibodies in xenograft models bearing HLA-E-expressing human cancer cells.^48^ Moreover, Previous clinical research validated the safety of combining anti-NKG2A with mAb-based immunotherapies.^49^ So, we performed the first clinical study to address a potential concern that the expression of NKG2A could inhibit NK cell cytotoxicity and, hence, prevent the implementation of this treatment strategy. Our results, however, clearly indicate that combining anti-NKG2A antibodies with NK cells is possible without significant adverse effects. 7 × 10^8^ NKG2A-treated NK cells were infused 3 times, consistent with other studies^50,51^ that reported minimal severe adverse effects with NK cell-based treatments. The transient symptoms such as fever and respiratory distress were observed and did not pose considerable safety concerns. In addition, hematological criteria like WBC, RBC, and platelet count were stable after NK cell administrations.

**Table 2:** Safety evaluation of the patients

**Patient**	**Fever**	**Rush**	**Chill**	**respiratory distress**	**Edema**	**Allergic signs**	**Headache**	**Arrhythmia**	**Safety assessment grading**
P-1	1	0	0	1	0	0	0	0	0
P-2	1	0	1	0	0	0	1	0	0
P-3	0	0	1	0	0	0	0	0	0

 Since our procedure does not provoke T cell proliferation, this could be a critical clinical advantage because it prevents the risk of GVHD in allogeneic NK cell therapy. However, the simultaneous administration of NKG2A mAb and NK cells is suggested to unleash patients’ NK and T cells to improve cytotoxic potential against intended cancer. Activated NK cells may not just directly kill tumor cells but also promote the expansion of T cells through the elimination of immune-suppressive cells and cytokine secretion.^52,53^

###  Clinical efficacy

 The clinical results are summarized in Table 3 and show that all 3 patients demonstrated stable disease (SD) after 1 month. Following 4 months, one patient (33.3%) remained in SD condition, but two patients (66.6%) exhibited progressive disease. Among three patients, two of them showed an increase in tumor size after 4 months.

**Table 3:** Tumor response

**Patients**	**Response**				**Response rate (%)** **(95 % CI)**	**Disease control rate (%) (95 % CI)**
	**CR**	**PR**	**SD**	**PD**		
				**1-month follow-up**		
n = 3	**-**	**-**	**3**	**0**	**0**	**100%**
				**4-months follow-up**		
n = 3	**-**	**-**	**1**	**2**	**0**	**33.3%**

CR: Complete Response, PR: Partial Response, SD: Stable Disease, PD: Progressive Disease

 In two studies, André et al and McWilliams et al demonstrated NK cell anti-cancer potential against cancer cells,^32,54^ corroborating our observation of enhanced cytotoxicity following anti-NKG2A treatment.

 In our survey, we attempted to examine the safety of pre-treated allogeneic NK cell therapy for treating HCC. These NK cells revealed a relatively high level of in vitro cytotoxicity which made them promising; however, we believed that it was more necessary to determine their toxicity before examining their efficacy. For this rationale, we chose a monotherapy where pre-treated NK cells were administered. To our surprise, anti-NKG2A combined with allogeneic NK cells for advanced HCC exhibited a stable disease response, and after 4 months, two patients experienced PD (Table 3).

###  Hematologic parameters

 The increase in WBC count is related to the high number of injected NK cells. The number of platelets remained consistent throughout the 48 hours after the last cell injection. The average INR ratio was 1.16 ± 0.3 on the first day of hospitalization, showing a non-significant increase 48 hours after the last NK cell infusion. The count of RBCs was in the normal range and showed no significant difference. The results indicated that our intervention had no notable effect on these values (Table 4).

**Table 4:** Descriptive data of laboratory parameters

**Patients**	**P-1**		**P-2**		**P-3**	
	**Before**	**After**	**Before**	**After**	**Before**	**After**
WBC	5.4	7.85	4.6	6.7	6.8	8.9
RBC	5.4	4.8	5.9	6.3	5.4	5.8
Platelet count	159	270	226	198	163	110
BUN	53.3	53.7	56.5	56.9	55.8	56.4
Cr	0.9	0.7	0.7	1.17	1.07	1.04
AST	97	90	89	87	132	98
ALP	205	237	214	223	265	276
CRP	9.3	8.7	8.4	9.8	9.2	8.7

###  Biochemical parameters

**Figure 4:**
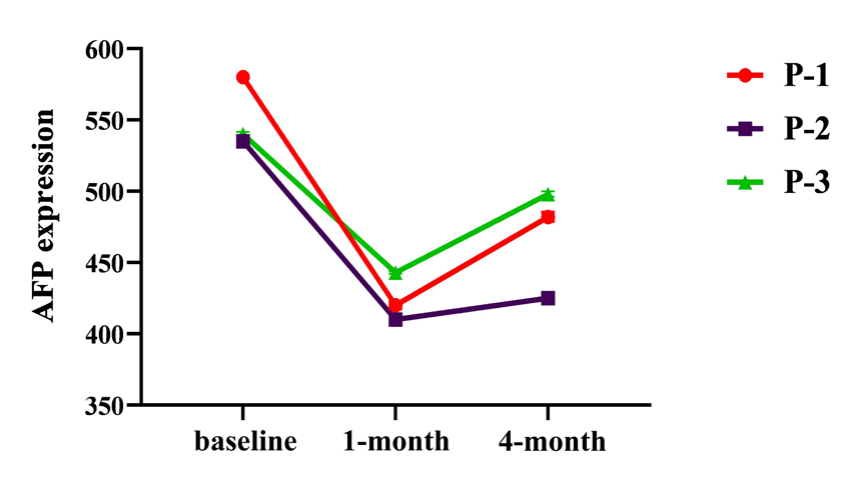
** Expression of AFP. **Administration of anti-NKG2A pretreated NK cells decreased AFP serum levels after 1 month in all 3 patients (not significant, *P* > 0.05). However, two patients showed an increase in their expression level after 4 months (*P* > 0.05)

 The daily measurement of BUN and Cr levels indicated that the injection of NK cells did not significantly affect these factors. Serum levels of liver factors, including ALP and ALT, remained stable throughout the 48 hours of follow-up. The results of CRP measurement demonstrated that the administration of NK cells did not affect the level of CRP during follow-up. Although AFP levels were above normal, NK cell injection decreased AFP serum levels after 1 month and increased after 4 months in 2 patients (Figure 4). Gong et al represented NKG2A gene deletion-induced NK cells better than its monoclonal antibody.^55^ However, after one month, NK cells caused a significant decrease in AFP, which slightly increased over the next three months.

## Conclusion

 Combining mAbs with therapy appears promising after confirming the safety of administering our pre-treated cells. Our combination approach’s relative merits and demerits compared to the standard regimens require further elucidation. Still, it is crucial to investigate any potential synergies between the T cell and NK cell infusion. This study’s results suggest that combining NK cells with other immunotherapies like anti-NKG2A can be feasible and well tolerated. NK cells do not cause GVHD without T cell receptors. This novel study makes using allogeneic “off-the-shelf” NK cell products easier from healthy donors, which could enhance NKG2A mAb response.

## Competing Interests

 All authors declare that they have no competing interests.

## Ethical Approval

 Not applicable.
